# Sinonasal Adenocarcinoma: Clinicopathological Characterization and Prognostic Factors

**DOI:** 10.7759/cureus.56067

**Published:** 2024-03-13

**Authors:** Marta Baptista Freitas, Mariana Costa, Andreia Freire Coelho, Pedro Rodrigues Pereira, Manuel Leal, Cristina Sarmento, Lúcia Águas, Miguel Barbosa

**Affiliations:** 1 Medical Oncology, Centro Hospitalar Universitário de São João, Porto, PRT; 2 Pathology, Centro Hospitalar Universitário de São João, Porto, PRT; 3 Otolaryngology - Head and Neck Surgery, Centro Hospitalar Universitário de São João, Porto, PRT

**Keywords:** protective occupational measures, induction chemotherapy, occupational risk exposure, p53 overexpression, colonic intestinal-type adenocarcinoma, papillary intestinal-type adenocarcinoma, intestinal-type adenocarcinoma, sinonasal adenocarcinoma

## Abstract

Sinonasal (SN) malignancies are rare. Within SN adenocarcinomas, the most frequent are intestinal-type adenocarcinomas (ITACs). ITAC has been associated with wood and leather dust occupational exposure and TP53 mutations. Not much information is available regarding its characterization and treatment. The aim of this study is to characterize the clinicopathologic and prognostic factors of patients with sinonasal adenocarcinomas (SNACs) treated in our tertiary-level hospital. A retrospective, consecutive study including SNAC patients diagnosed between 2004-2023 was conducted. Clinicopathological data was collected, and p53 status was assessed in the tumor specimens. The association between p53 status and clinicopathological variables, as well as their impact on survival, was evaluated.

In total, 35 were included, most of them having ITAC (91.4%) with papillary subtype (37.5%); the majority were subjected to occupational risk exposure (82.9%). Overexpression of p53 was identified in 48.6% of the tumors. Papillary and colonic subtypes were associated with higher median progression-free survival (mPFS) than mucinous and solid subtypes (mPFS 37 months, 95% CI, 20.0-54.0, vs. 9 months, 95% CI, 7.15-10.85, p=0.01); the former was also associated with higher median overall survival (mOS) (mOS 64 months, 95% CI, 37.18-90.81 vs. 14 months, 95% CI, 0-41.58, p=0.02). Histologic grade 1-2 and macroscopic complete resection were associated with higher PFS (PFS of five months of 90.9% vs. 33.3%, p=0.01; mPFS of 37 months, 95% CI, 4.93-69.07 vs. 10 months, 95% CI, 6.43-13.57, p=0.04, respectively). Disease recurrence with distant metastases was associated with lower OS (11 months, 95% CI, 6.1-15.9 vs. 53 months, 95% CI, 22.70-83.30, p=0.04). This study reinforces the importance of protective occupational measures. Future studies will be important to validate the best treatment strategy in the advanced stages of this disease and also to identify new prognostic and/or therapeutic target biomarkers in SNAC.

## Introduction

Sinonasal adenocarcinomas (SNACs) are rare and heterogeneous tumors, corresponding to around 3% of upper aerodigestive tract malignant tumors [1-3]. They are more prevalent in males and are associated with poor overall survival (OS), as they are often diagnosed at an advanced stage when there are fewer therapeutic options [4]. Sinonasal (SN) tumors include epithelial and non-epithelial subtypes. In the epithelial subgroup, squamous cell carcinoma accounts for around 80% of these tumors [1]. Adenocarcinomas (10-20%) are the second most common subtype of SN epithelial tumors, the most common being intestinal-type adenocarcinoma (ITAC) [1-3, 5]. ITAC is often related to occupational exposure to wood and leather dust (both the intensity of exposure and its duration), and it can be diagnosed up to 40 years after exposure [1-3, 5]. A Portuguese study, which included 39 patients with ITAC, also identified cork occupational exposure as a risk factor for ITAC [6]. In this study, 69% of patients with ITAC had a history of prolonged occupational exposure to wood dust, cork, polycyclic aromatic hydrocarbons, formaldehyde, coal, isomastic material, or the textile industry [6].

Regarding the genetic features of ITAC, mutations in the TP53 tumor suppressor gene are the most common genetic alterations in these tumors, although its incidence varies greatly between studies, ranging from 18-86% [3, 4, 7]. TP53 gene mutation may lead to p53 protein overexpression [3, 8]. These alterations seem to be related to occupational exposure to wood dust and may result in higher-grade tumors [3].

ITAC treatment depends on its location and stage, as well as patient-related factors [1]. In general, these tumors are treated with surgery and radiotherapy (RT), in some cases with the addition of chemotherapy (CT). In the earliest stages, surgery is the treatment of choice whenever feasible [1]. Depending on the stage and location, adjuvant RT may be recommended for better local control, and, in some cases, adjuvant chemoradiotherapy (CRT) may be considered [1]. The five-year survival rate is around 60-70%, varying according to the grade, stage, and type of treatment that the patient received [2, 9]. Patients unable to undergo surgery, who refuse it, or in selected cases, definitive RT or CRT may be treatment options [1]. In selected cases at an advanced stage, neoadjuvant CT can play an important role [1]. Phase II clinical trials have shown response rates between 54-60% with induction CT (all subtypes of SNAC), with PFS and OS at five years of 38% and 46%, respectively [10, 11]. These studies also concluded that response to induction CT predicts a better prognosis [10, 11].

SNAC has high recurrence rates (50-60%), most of which are local recurrences [1]. Data on salvage therapy is scarce, with an estimated survival rate of 17% at five years [1].

Many patients living in locations with several wood industries in their area are treated in our hospital. Therefore, considering the well-documented causal association between such occupational risk exposure and the development of ITAC, and the need to better characterize these rare tumors, we aim to analyze the clinicopathological features and identify prognostic factors of SNAC patients treated in our tertiary-level hospital.

## Materials and methods

Study design and patients

We conducted a retrospective cohort analysis of all consecutive patients with SNAC, diagnosed from 01/01/2004 to 01/06/2023 and treated at the Centro Hospitalar Universitário de São João (CHUSJ), a Portuguese tertiary-level hospital. The cut-off date for follow-up was 31/11/2023.

Data concerning demographics, occupational risk exposure, tumor stage, location, type of treatments performed (surgery, RT, CT as single treatments or in combination as a multimodal treatment), and oncological outcomes were collected by consulting the patients' medical records.

The study was carried out following the ethical principles of the 1964 Declaration of Helsinki, as well as European data protection legislation. The study was approved by our institutional ethics committee (approval reference: CE 323/2023).

Inclusion criteria

The inclusion criteria were adult patients with histologically confirmed primary SNAC, who were diagnosed from 01/01/2004 to 01/06/2023 and underwent surgery, RT and/or CT in CHUSJ.

Exclusion criteria

Patients with SN malignancies other than SNAC or without available information regarding tumor and treatment features in the electronic clinical records were excluded from the study.

Clinical samples

Tumors were re-staged according to the American Joint Committee on Cancer (AJCC) TNM staging, 8th edition, 2017 [12]. The p53 status was assessed using IHC methods and it was evaluated by an experienced surgical pathologist, regarding the percentage of tumor cells with nuclear immunoreactivity for p53 (0-100%). Patients were then divided into two groups considering p53 tumor expression: null-low expression (if tumor cells with nuclear immunoreactivity for p53 < 50%) and overexpression (tumor cells with nuclear immunoreactivity for p53 ≥ 50%).

Data analysis

The chi-square test or Fisher's exact test, as appropriate, was used to assess the association between p53 status and clinicopathological variables. OS was defined as the time between diagnosis and death from any cause or to the last medical visit. The SLP refers to the time between diagnosis and recurrence/ progression or the last follow-up visit. Survival analysis was performed using the Kaplan-Meier method and univariate analysis was performed using the log-rank test. Differences were considered statistically significant if p < 0.05. Statistical analyses were performed using Statistical Package for the Social Sciences version 25 (IBM Corp., Armonk, NY).

## Results

In total, 35 patients with SNAC were included, with a median age at diagnosis of 61 (42-81) years, mostly male (n=34, 97.1%) and with ECOG PS between 0 and 1 (n=31, 88.6%). The most frequent primary location of the tumor was the nasal cavity (n=18, 51.4%) and the most common histological type was ITAC (n=32, 91.4%); the papillary subtype was present in 37.5% (n=12) of the patients. Overexpression of p53 was identified in 48.6% (n=17) of the tumors, 42.9% were diagnosed at an advanced stage (IVA-C), with orbital involvement in 45.7% of cases. Risk occupational exposure was identified in around 83% of patients, mostly from exposure to wood dust (77.1%). The clinicopathological features of the patients included are described in Table 1.

**Table 1:** Patients’ clinicopathological features *Lannetti-Valentini classification AJCC: American Joint Committee on Cancer; ECOG PS: Eastern Cooperative Oncology Group Performance Status; ITAC: intestinal-type adenocarcinoma

Clinicopathological features (n=35)		n (%)
Age	≤ 60 years old	15 (42.9)
	> 60 years old	20 (57.1)
ECOG PS	0	19 (54.2)
	1	12 (34.3)
	≥ 2	3 (8.6)
	Unknown	1 (2.9)
Occupational risk exposure	Yes	29 (82.9)
	No	4 (11.4)
	Unknown	2 (5.7)
Type of occupational risk exposure (n=29)	Wood dust	27 (77.1)
	Other	2 (5.7)
Smoking ≥20 pack years	Yes	13 (37.1)
	No	16 (45.7)
	Unknown	6 (17.1)
Medium-high risk alcohol consumption	Yes	14 (40.0)
	No	14 (40.0)
	Unknown	7 (20.0)
Charlson Comorbidity Index	≤ 5	23 (65.7)
	> 5	10 (28.6)
	Unknown	2 (5.7)
Clinical stage - AJCC 8^th^ ed.	I-II	11 (31.4)
	III	9 (25.7)
	IVA-IVB	14 (40.0)
	IVC	1 (2.9)
Orbital invasion*	Yes	16 (45.7)
	Grade 1	4 (25.0)
	Grade 2	4 (25.0)
	Grade 3	7 (43.7)
	No	18 (51.4)
	Unknown	1 (2.9)
Visual impairment at diagnosis	Yes	10 (28.6)
	No	24 (68.6)
	Unknown	1 (2.9)
ITAC	Yes	32 (91.4)
	No	3 (8.6)
Histological subtype of ITAC (n=32)	Papillary	12 (37.5)
	Colonic	9 (28.1)
	Mucinous	9 (28.1)
	Solid	2 (6.3)
Tumor regions with cribriform architecture	Yes	9 (25.7)
	No	26 (74.3)
Tumor localization	Ethmoid sinus	16 (45.7)
	Maxillary sinus	1 (2.9)
	Nasal cavity	18 (51.4)
Histologic grade	Grade 1	3 (8.6)
	Grade 2	9 (25.7)
	Grade 3	3 (8.6)
	Unclassified	20 (57.1)
p53 overexpression	Yes	17 (48.6)
	No	18 (51.4)

Regarding treatment, 54.3% underwent multimodal treatment, which was the most common surgery followed by adjuvant RT (n=14, 40.0%). More than 90% of the patients (n=32, 91.4%) underwent surgery and 43.7% (n=14) of them also underwent adjuvant RT. Macroscopically complete resection was achieved in 71.9% of the surgical cases and the orbit was preserved in 85.7% of them. Only 25% of the patients who underwent surgery had surgical margins with no evidence of disease (R0); in 43.7% of cases, it was not possible to assess the margins (Rx), mainly due to fragmentation of the specimen. The vast majority of patients (90.9%) who did CT (induction or palliative CT; neoadjuvant, radical or adjuvant CRT, n=11) as part of their treatment plan received a platinum-based regimen, either in monotherapy as a radiosensitizer or in combination with fluoropyrimidines. Of the four patients who underwent radical CRT, neoadjuvant CRT, or induction CT followed by surgery, two (50%) had a partial response to treatment and the other two (50%) had disease progression. The median follow-up time was 18 (1-100) months. In total, 16 patients (45.7%) had disease recurrence or progression during follow-up time, and almost half of them underwent surgery (n=7, 43.7%). Table 2 systematizes the treatment details (initial treatment and after disease relapse/progression).

**Table 2:** Association between p53 tumor expression and clinicopathological and treatment features *Lannetti-Valentini classification AJCC: American Joint Committee on Cancer, ECOG PS: Eastern Cooperative Oncology Group Performance Status, CT: chemotherapy

Treatment characteristics (n=35)		n (%)
Multimodal treatment	Yes	19 (54.3)
	No	16 (45.7)
Type of treatment	Surgery	9 (25.7)
	Surgery + RT	14 (40.0)
	Surgery + CRT	6 (17.1)
	CRT + surgery	2 (5.7)
	CT + surgery	1 (2.9)
	Radical CRT	1 (2.9)
	Palliative CT	1 (2.9)
	Best supportive care	1 (2.9)
Type of surgical resection (n=32)	Macroscopic complete	23 (71.9)
	Incomplete	9 (28.1)
Orbital exenteration (n=32)	Yes	2 (5.7)
	No	30 (85.7)
Pathological stage - AJCC 8th ed. (n=32)	I-II	7 (21.9)
	III	8 (25.0)
	IVA-IVB	12 (37.5)
	Unclassified	5 (14.3)
Complications after surgery (n=32)	Yes	10 (31.2)
	No	17 (53.1)
	Unknown	5 (14.3)
Platinum-based CT regimen (n=11)	Yes	10 (90.9)
	- Cisplatin + 5-FU	3 (27.3)
	- Cisplatin	6 (54.5)
	- Carboplatin	1 (9.1)
	No	1 (9.1)
Treatment response (n=4)	Partial	2 (50.0)
	Progression	2 (50.0)
Disease relapse/progression	Yes	16 (45.7)
	No	19 (54.3)
Disease progression with distant metastasis (n=16)	Yes	4 (25.0)
	No	12 (75.0)
Distant metastasis location (n=4)	Bone	2 (50.0)
	Lung	1 (25.0)
	Bone and lung	1 (25.0)

Treatment with surgery alone was significantly more common in the group of patients with null-low p53 expression (p=0.01), while the other clinicopathological characteristics showed no statistically significant association with p53 status (Table 3). It was not possible to analyze the association between p53 expression and types of CT treatments (induction CT, radical CRT, neoadjuvant, or adjuvant CRT) due to the small number of patients who underwent each modality.

**Table 3:** Association between p53 tumor expression and clinicopathological and treatment features *Lannetti-Valentini classification AJCC: American Joint Committee on Cancer, ECOG PS: Eastern Cooperative Oncology Group Performance Status, ITAC: intestinal-type adenocarcinoma

Clinicopathological and treatment features (n=35)		p53 null – low expression (n=18)	p53 overexpression (n=17)	p-value
Age	≤ 60 years old	7	8	0.44
	> 60 years old	11	9	
ECOG PS (n=34)	0	11	8	0.38
	≥ 2	7	8	
Occupational risk exposure (n=33)	Yes	14	15	0.07
	No	4	0	
Type of occupational risk exposure (n=29)	Wood dust	13	14	0.74
	Other	1	1	
Smoking ≥20 pack years (n=29)	Yes	6	7	0.20
	No	11	5	
Medium-high risk alcohol consumption (n=28)	Yes	8	6	0.65
	No	8	6	
Charlson Comorbidity Index (n=33)	≤ 5	10	13	0.15
	> 5	7	3	
Clinical stage - AJCC 8^th^ ed.	I-II	7	4	0.26
	III	4	5	
	IV	7	8	
Orbital invasion* (n=34)	Yes	8	8	0.51
	No	10	8	
Visual impairment at diagnosis (n=34)	Yes	5	5	0.56
	No	13	11	
ITAC	Yes	16	16	0.52
	No	2	1	
Histological subtype of ITAC (n=32)	Papillary and colonic	10	11	0.50
	Others	6	5	
Tumor regions with cribriform architecture	Yes	5	4	0.50
	No	13	13	
Tumor localization	Perinasal sinus	9	8	0.56
	Nasal cavity	9	9	
Histologic grade (n=15)	Grade 1-2	6	6	0.55
	Grade 3	2	1	
Multimodal treatment	Yes	7	12	0.06
	No	11	5	
Type of treatment	Upfront Surgery	8	1	0.01
	Others	10	16	
Disease relapse/progression	Yes	10	6	0.19
	No	8	11	
Disease progression with distant metastasis (n=16)	Yes	2	2	0.49
	No	8	4	

**Figure 1:**
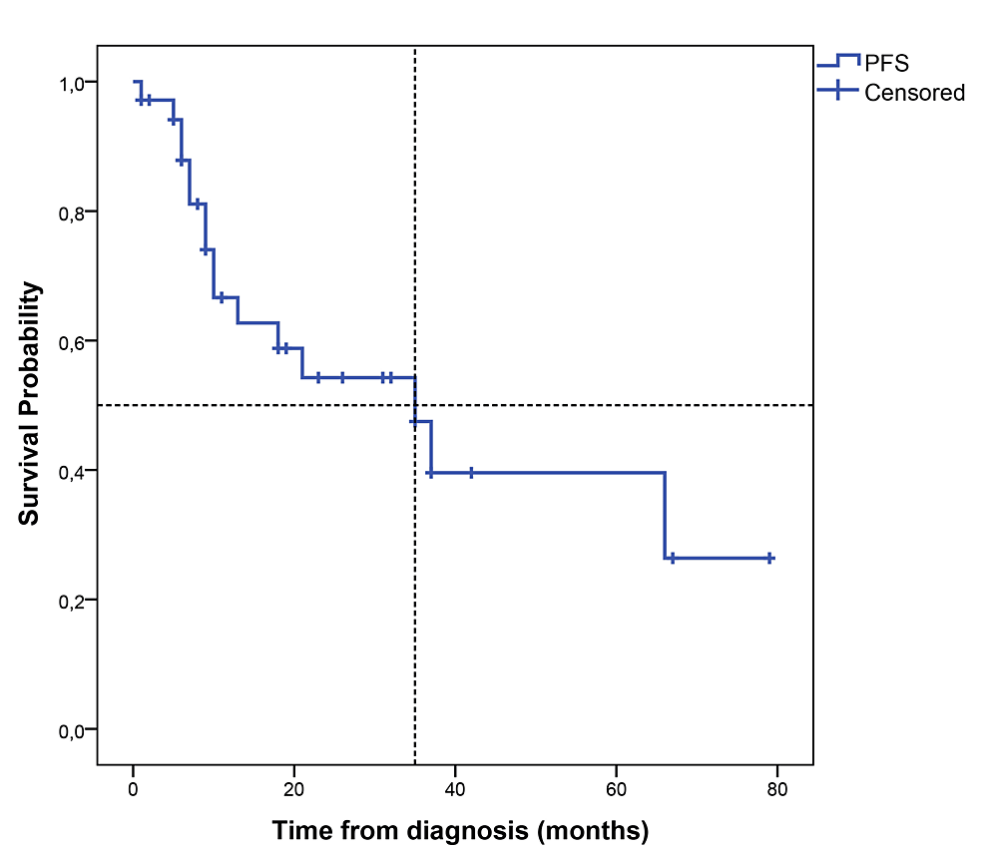
Kaplan-Meier curve for PFS PFS: progression-free survival

**Figure 2:**
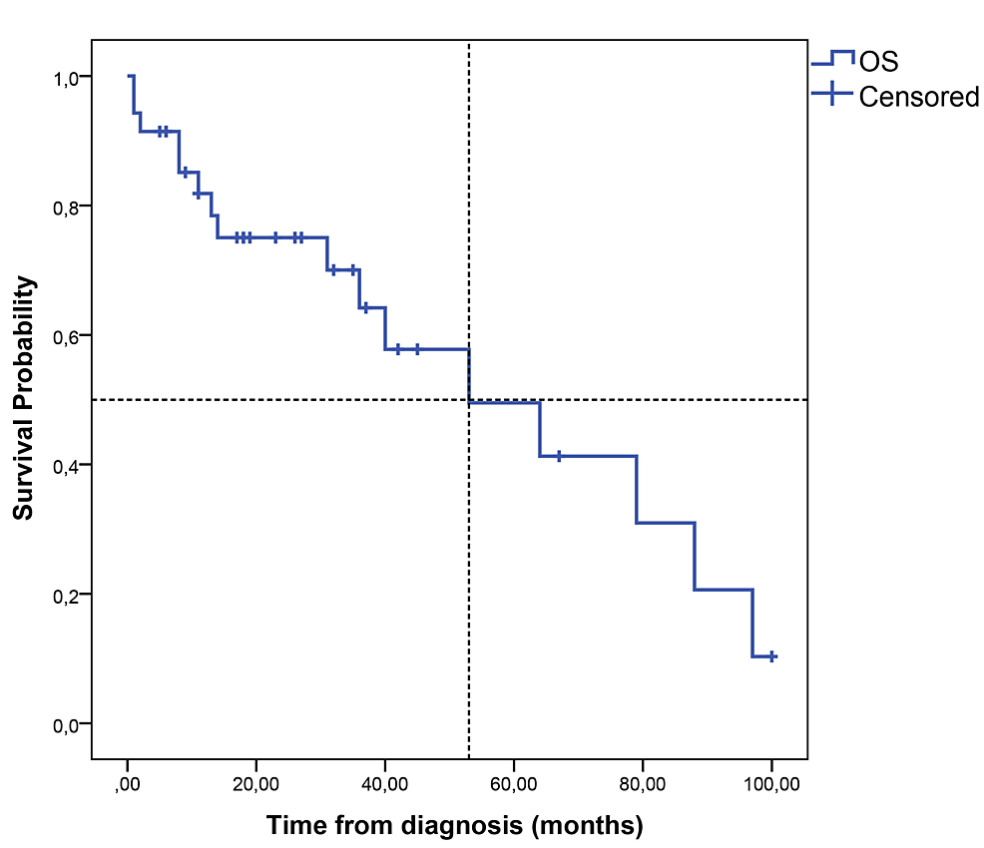
Kaplan-Meier curve for OS OS: overall survival

**Figure 3:**
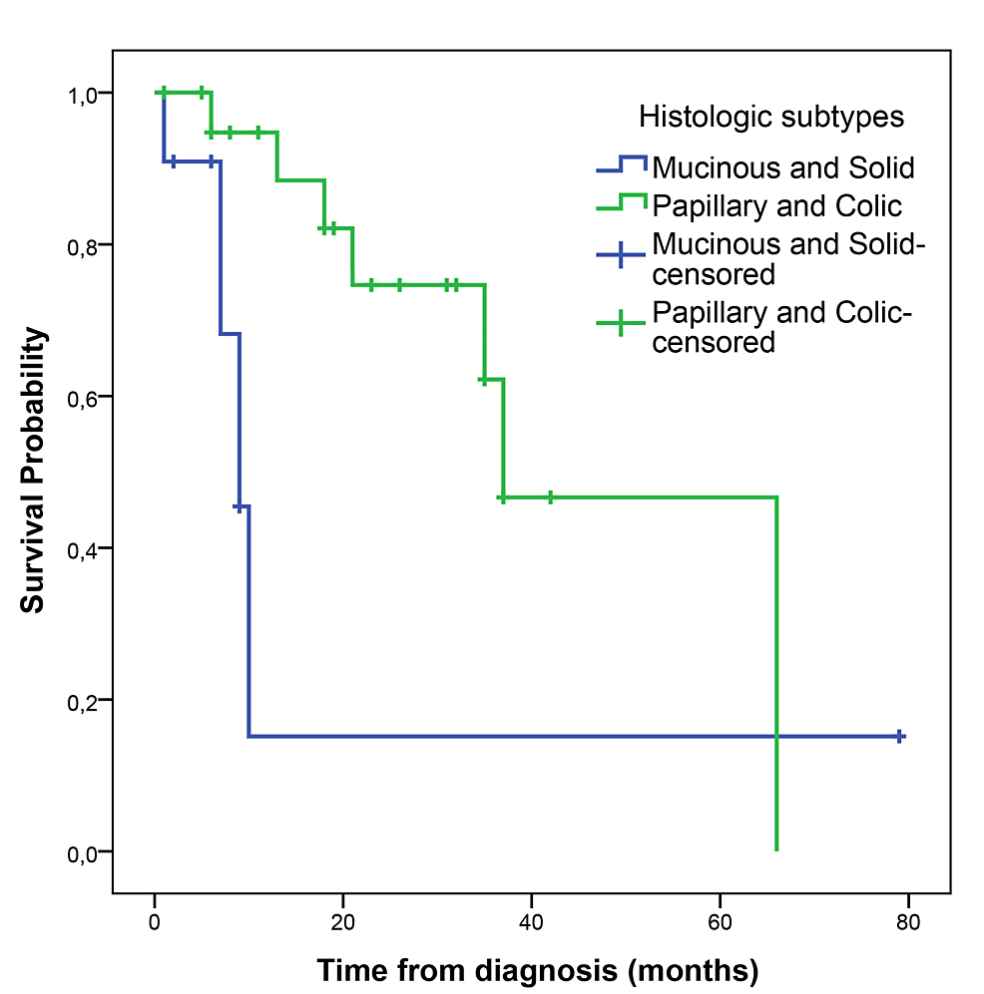
Kaplan-Meier curve for PFS according to histologic ITAC subtype PFS: progression-free survival; ITAC: intestinal-type adenocarcinoma

**Figure 4:**
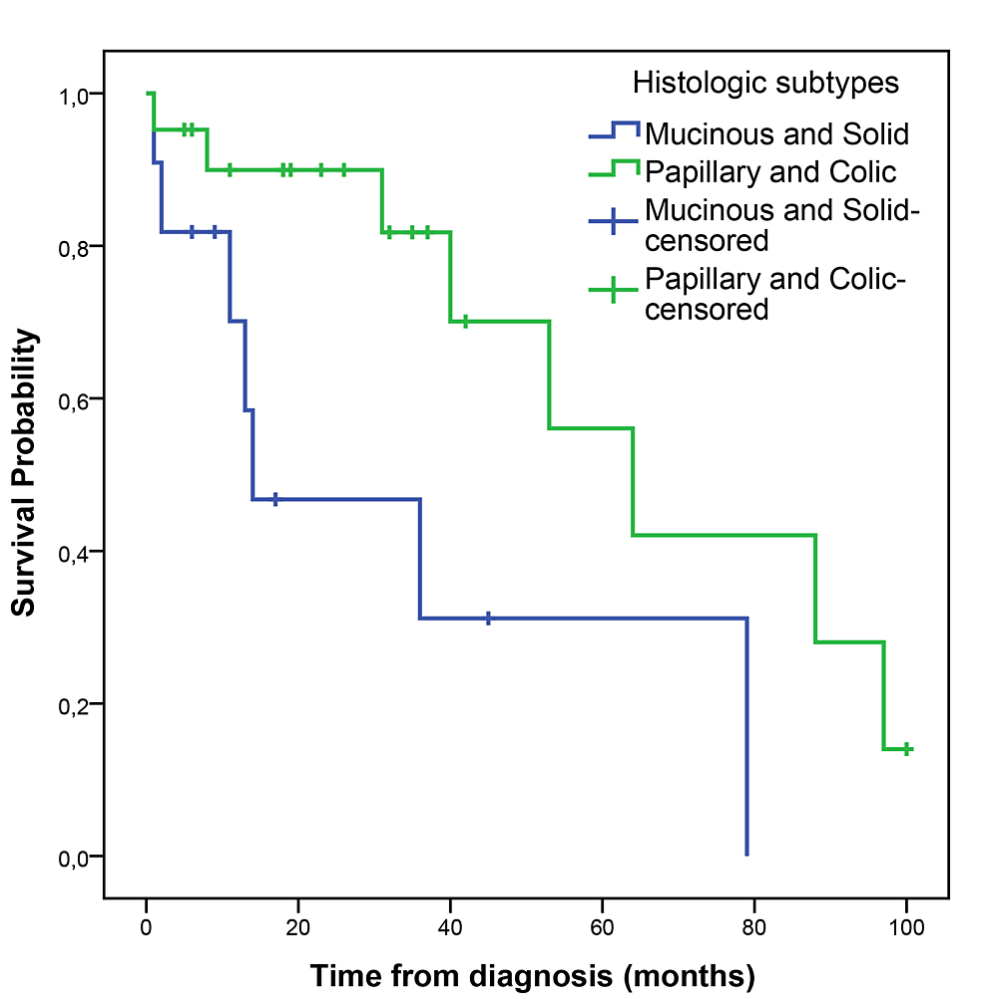
Kaplan-Meier curve for OS according to histologic ITAC subtype OS: overall survival; ITAC: intestinal-type adenocarcinoma

The median follow-up time was 23 (1-100) months. The median PFS (mPFS) was 35 months (95% CI, 14.58-55.41), with a five-year PFS of 39.5% (Figure 1). The median OS (mOS) was 53 months (95% CI, 19.07-86.93), with 60 months of 50% (Figure 2). The papillary and colonic subtypes, when compared to the other subtypes, were associated with better PFS (mPFS: 37 months, 95% CI, 20.0-54.0, vs. 9 months, 95% CI, 7.15-10.85, p=0.01) and better OS (mOS 64 months, 95% CI, 37.18-90.81, vs. 14 months, 95% CI, 0-41.58, p=0.02) (Figures 3-4).

Histologic grade 1-2 and surgery with macroscopic complete resection were associated with better PFS (5-month PFS, 90.9% vs. 33.3%, p=0.01; mPFS 37 months, 95% CI, 4.93-69.07, vs. 10 months, 95% CI, 6.43-13.57, p=0.04, respectively). Disease relapse/progression with distant metastasis was shown to be significantly associated with lower OS: patients with distant metastases had a mOS of 11 months (95% CI, 6.1-15.9) compared to 53 months (95% CI, 22.70-83.30) in those with local disease recurrence/progression (p=0.04). Although not statistically significant, grade 1-2 compared to grade 3 tumors showed a trend towards better OS (40 months, 95% CI, 10.56-69.44, vs. 8 months, 95% CI, 0-19.20, p=0.06). Table 4 shows the association analysis between the survival outcomes and the study population characteristics (univariate analysis).

**Table 4:** Association between population characteristics and survival outcomes (PFS and OS) ECOG PS: Eastern Cooperative Oncology Group Performance Status; ITAC: intestinal-type adenocarcinoma; PFS: progression-free survival; OS: overall survival

Clinicopathological and treatment features (n=35)		mPFS (95% CI) (months)	p-value	mOS (95% CI) (months)	p-value
Age	≤ 60 years old	21 (4.4-37.58) vs. 66 (8.77-123.22)	0.24	88 (16.32-159.68) vs. 53 (18.14-87.86)	0.65
	> 60 years old				
ECOG PS	0	37 (32.59-41.41) vs. 10 (8.51-11.48)	0.15	53 (27.93-78.06) vs. 36 (0.40-104.48)	0.26
	≥ 2				
Occupational risk exposure	Yes	20 (0-49.85) vs. 35 (14.72-55.28)	0.50	53 (14.23-91.77) vs. 36 (0-77.91)	0.78
	No				
Smoking ≥20 pack years	Yes	12-month PFS 48.6% vs. 46.9%	0.95	36 (25.67-46.33)vs. 88 (27.92-148.07)	0.12
	No				
Charlson Comorbidity Index	≤ 5	21 (0-49.61) vs. 66 (0-148.94)	0.59	36 (26.34-45.66) vs. 88 (18.14-157.86)	0.22
	> 5				
Histological subtype of ITAC	Papillary and colonic	37 (20.0-54.0) vs. 9 (7.15-10.85)	0.01	64 (37.18-90.81) vs. 14 (0-41.58)	0.02
	Others				
Tumor regions with cribriform architecture	Yes	35 (20.36-49.64) vs. 21 (0-57.29)	0.93	88 (26.31-149.69) vs. 40 (11.23-68.77)	0.40
	No				
Tumor localization	Perinasal sinus	13 (8.51-17.49) vs. 66 (19.71 –112.30)	0.08	64 (17.11-110.89) vs. 53 (27.85-78.15)	0.83
	Nasal cavity				
Histologic grade	Grade 1-2	5-month PFS 90.9%vs. 33.3%	0.01	40 (10.56-69.44)vs. 8 (0-19.20)	0.06
	Grade 3				
p53 overexpression	Yes	37 (6.9-67.03) vs. 35 (0-81.71)	0.69	64 (14.77-113.23) vs. 53 (14.78-113.23)	0.97
	No				
Multimodal treatment	Yes	24-month PFS 57.7% vs. 49.2%	0.51	64 (28.29-99.71) vs. 53 (22.87-83.12)	0.83
	No				
Type of treatment	Upfront Surgery	24-month PFS 55.6% vs. 53.9%	0.48	88 (17.52-158.48) vs. 40 (14.96-65.04)	0.10
	Others				
Type of surgical resection	Macroscopic complete	37 (4.93-69.07) vs. 10 (6.43-13.57)	0.04	9-month OS 86.1% vs. 68.6%	0.60
	Incomplete				
Complications after surgery	Yes	10 (4.30-15.70) vs. 63 (34.42-97.58)	0.13	36 (0-76.4) vs. 53 (43.4-150.60)	0.10
	No				
Disease progression with distant metastasis	Yes	-	-	11 (6.1-15.9) vs. 53 (22.70-83.30)	0.04
	No				

## Discussion

In this study, the majority of patients diagnosed with SNAC were males (97.1%), older than 60 years old (57.1%), with a high-risk occupational exposure (82.9%) identified and the most frequent type of SNAC was ITAC (91.4%), which is in accordance to current literature data [3, 5, 13]. However, this high number of cancers in the male population could be influenced by the fact that woodworkers are mainly male. The most common histological subtype in this population was papillary (37.5%) and not colonic as usually described [5]. There was also a higher prevalence of mucinous tumors (28% vs. 14%) and a lower prevalence of solid tumors (6.3 vs. 20%) than described in the literature [5]. The most prevalent primary location was the nasal cavity (51.4%), followed by the ethmoid sinus (47.5%). Although the ethmoid sinus has been described in the literature as the most frequent primary location of ITAC, several other studies have reported the nasal cavity as the most common primary location of ITAC, as in our study [5, 6, 9, 14]. Regarding the stage at diagnosis, 40% of the patients were diagnosed at an advanced stage (IVA-B) and 1 patient already had distant metastases. Orbital involvement was found in 45.7% (Grade 3 in 43.7% of these).

Almost half of the tumors in this study (48.6%) had p53 overexpression, in agreement with the data reported in other studies [3, 7]. Genetic alterations in the TP53 gene are a common event in head and neck carcinomas and occur in the early stages of carcinogenesis [7, 15]. These alterations are also very prevalent in SNAC, especially when there is intense and prolonged exposure to wood dust [7, 15]. In our population, p53 status was not significantly correlated with wood dust occupational exposure. Several studies underline that changes in the TP53 gene are independent of smoking exposure, which corroborates the findings in our population, in which patients’ moderate- to high-risk smoking habits were not associated with p53 overexpression [5, 7, 15]. Additionally, p53 expression seems to play a role in the response to treatment [5]. Studies showed that ITAC with functional p53 (wild-type or the mutated protein with normal or enhanced function) CT treatment was more effective than in tumors without a functional protein, which suggested the status of p53 as a potential biomarker for the therapeutic decision in ITAC [5, 7]. However, phase I and II studies have shown that even ITAC with p53 overexpression did not respond as expected to induction CT, unlike undifferentiated carcinomas and those with neuroendocrine differentiation that had a better response rate [10, 11]. In our study, few patients were submitted to induction CT, so this analysis couldn’t be carried out. We found that null-low p53 expression was associated with treatment with surgery alone, despite the associations between p53 status and tumor stage and grade were not statistically significant. The small sample size may have influenced these results. It should also be pointed out that p53 overexpression seems to be less frequent in mucinous ITAC, with around 33% in this study, which is in line with the results described in the literature [7].

Along with the tumor histopathological features, the tumor stage, and the patient's ECOG PS are key factors in the decision of the best therapeutic approach for each patient. Surgery continues to play a very important role in the treatment of these tumors [5]. Technical advances have made surgery safer, with fewer complications, more effective regarding oncological outcomes, and capable of providing a better quality of life for the patient [5]. It is still the treatment of choice for local disease control in cases of resectable tumors [16]. In our population, the vast majority of patients underwent upfront surgery: 25.7% have done only surgery and 40% underwent surgery followed by adjuvant RT. Macroscopical complete resection was achieved in 71.9% of the cases and the orbit was preserved in 85.7%.

Given the rarity of this type of tumor, there are no randomized clinical trials that clearly define adjuvant treatments [5]. There is sustained evidence of survival benefits when adjuvant RT is employed in cases of positive margins, advanced-stage tumors (pathological T3-4), and poorly differentiated ITACs [5, 16]. It should be noted that in this population, only 25% of the patients submitted to surgery had R0 surgical margins due to the high percentage of cases (34.4%) in which it was not possible to determine it, either because of specimen fragmentation or tumor location. Concerning adjuvant CT, there is some evidence of its benefits in combination with RT (cisplatin-based regimens) in cases of positive surgical margins and persistent disease in unresectable tumors due to their localizations [5]. In our sample, six patients underwent adjuvant CRT, five with cisplatin concomitant with RT, and one with weekly carboplatin (for better tolerance, due to ECOG PS). As a result, around half (54%) of the patients in the study underwent multimodal treatment, the most common being a combination of surgery followed by adjuvant RT (40.0%). Of the patients who underwent induction CRT, one of them was treated with cisplatin monotherapy and another with cisplatin combined with fluoropyrimidines (CF). CF regimen was also used in the case of induction QT followed by surgery and in the patient that did palliative CT since the diagnosis. This CT regimen has also been reported in other SNAC studies and is associated with objective responses [17, 18]. 

In total, 16 patients (45.7%) had disease recurrence or progression during the follow-up period; four (25%) of them had distant metastases. ITAC has a high recurrence rate, especially in the case of local disease recurrence (around 88%) [5, 17]. Therefore, in many cases, it is possible to treat the recurrence with surgery or radiotherapy, which was the case in 56.2% of the patients of the study [5, 17]. In total, 16 patients had a relapse/progression of the disease, with a mPFS of 35 months (95% CI, 14.58-55.41), with a five-year PFS of 39.5%, lower than the most recent data in the literature (five-year PFS of 72.3%) [5]. The mOS was 53 months (95% CI, 19.07-86.93), with a five-year-OS of 50%, also lower than survival data reported in the literature (five-year OS between 72.7-54%) [5, 16]. In our study, papillary and colonic subtypes were associated with better PFS (mPFS of 37 months, 95% CI, 20.0-54.0, vs. 9 months, 95% CI, 7.15-10.85, p=0.01) and better OS (mOS of 64 months, 95% CI, 37.18-90.81, vs. 14 months, 95% CI, 0-41.58, p=0.02), which is in line with previous studies that have shown that papillary tumors are associated with a better prognosis while solid and mucinous subtypes are associated with worse survival outcomes [19]. Both lower histological grade (grade 1-2) and macroscopic complete resection were associated with better PFS (5-month PFS 90.9% vs. 33.3%, p=0.01; mPFS 37 months, 95% CI, 4.93-69.07, vs. 10 months, 95% CI, 6.43-13.57, p=0.04, respectively). Although not statistically significant, grade 1-2 compared to grade 3 tumors showed a trend towards better OS (40 months, 95% CI, 10.56-69.44, vs. 8 months, 95% CI, 0-19.20, p=0.06), which is in line with published data, in which a higher grade is associated with worse survival outcomes [14, 16, 19]. Disease recurrence with distant metastases was also shown to be significantly associated with worse overall survival outcomes in this study (mOS of 11 months, 95% CI, 6.1-15.9, vs. 53 months, 95% CI, 22.70-83.30, p=0.04), as reported in previous studies [16].

It should be noted that this is a retrospective study, during a long period of time (around 20 years, with changes in the diagnosis and treatment guidelines of these tumors) and with a small sample size. All these factors are limitations of this study to be considered when analyzing the results. The lack of information on some of the parameters assessed in the older cases was also a major limitation of this analysis.

Despite these limitations, this study reinforces the importance of protective measures regarding occupational risk exposure. Based on these data, a public health intervention in the most affected regions is being planned, in order to increase health literacy among this population at higher risk of ITAC.

Future studies (ideally prospective and randomized) will be important to further evaluate the role of induction CT followed by surgery compared to surgery combined with adjuvant CRT in SNAC. Also, further studies will be crucial to clarify the role of p53 tumor status as a biomarker in this disease (evaluating its role as a possible prognostic biomarker and even predictive of treatment response).

## Conclusions

Despite the limitations of this study, it confirms the trend towards a higher incidence of ITAC in men, mainly associated with occupational exposure to wood dust in this sample. The prevalence of p53 overexpression was 48.6% and was not associated with any tumor clinicopathological features or survival outcomes, which can be due to the sample size of this study; this should be evaluated in future studies. Papillary and colonic subtypes, histological grade 1-2, and macroscopic complete resection were associated with better PFS. Papillary and colonic subtypes were also associated with better OS while distant metastases at disease recurrence were associated with worse OS.

This study reinforces the importance of protective occupational measures and the need for public health interventions to increase health literacy. Future studies will be important to validate the best treatment strategy in the advanced stages of this disease, the potential role of neoadjuvant treatment in these cases, and also to research new prognostic and/or therapeutic target biomarkers in SNAC.
